# Cerebral air emboli

**Published:** 2013

**Authors:** Abdorreza Naser Moghadasi, Omid Sanaei

**Affiliations:** 1 Sina Multiple Sclerosis Research Center, Sina Hospital, Tehran University of Medical Sciences, Tehran, Iran; 2 Department of Internal Medicine, North-Khorasan University of Medical Sciences, Shirvan, Iran

**Keywords:** Air Emboli, Chest Trauma, Brain CT Scan

## Case Report

**Figure 1:**
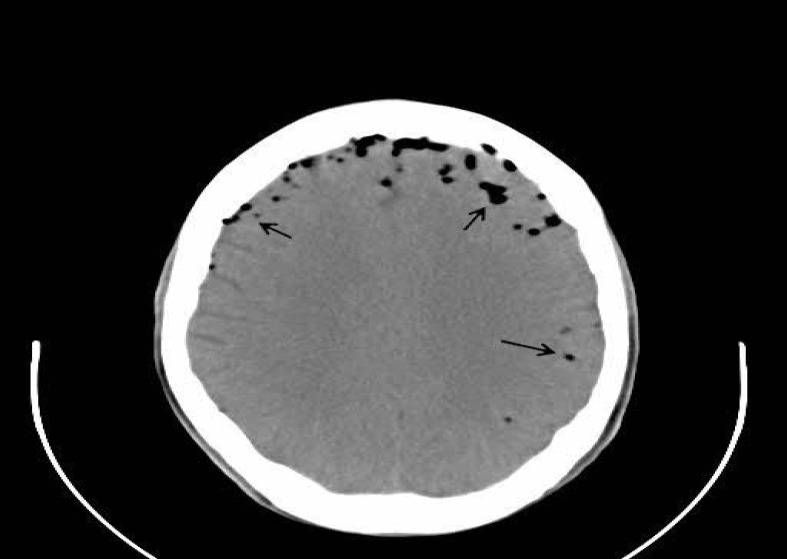
There were large amounts of air bubbles in the brain CT scan

**Figure 2:**
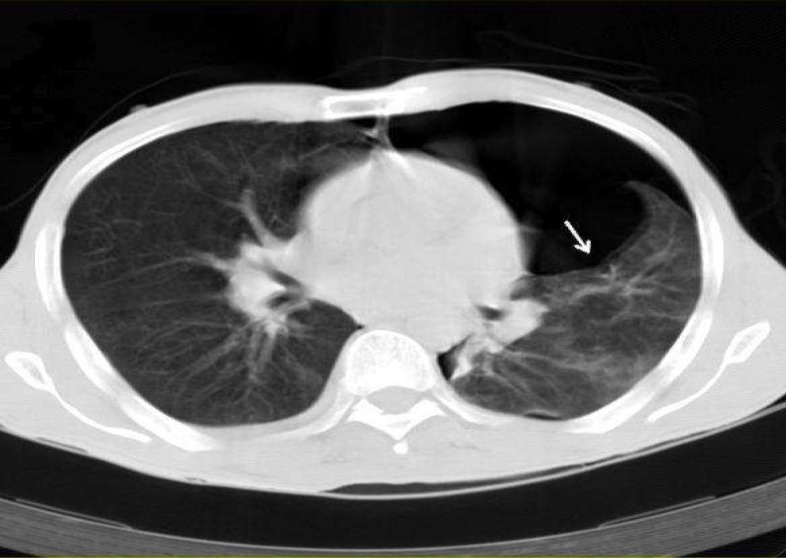
Chest CT scan uncovered a tension pneumothorax

A 30 year-old man referred to the emergency department with loss of consciousness and a stab wound in the right para-spinal region at about the level of the T6 vertebra. On admission, the patient was confused, agitated, and had automatism in his lips and mouth. There were no obvious signs of head trauma. Vital signs were stable, and lung and abdominal exams were normal. The brain computed tomography (CT) scan showed large amounts of air in it (Figure 1). Thus, we transferred the patient to the intensive care unit (ICU) and administered high doses of oxygen (10 Liter/min) with mask, while laying him in the trendelenburg position. Consciousness of the patient improved rapidly with these treatments. Although primary lung examination revealed no abnormal findings, the patient gradually went into respiratory distress. A lung CT scan uncovered that a tension pneumothorax has been developed (Figure 2). A subsequent thoracotomy was performed, during which tearing of a large branch of the left bronchial tree was detected and sutured. A thoracostomy tube was then established to evacuate the excess air from the pleural cavity. A second brain CT scan showed that all the air bobbles were gone. After one week, the patient was discharged in good condition. In this case, after bronchial tear, intra-pleural pressure was increased and led the air to pulmonary veins, the heart, aorta and finally the cranial arteries.

Cerebral air embolism is a threatening condition that could be caused by isolated penetrating chest injury.^1^ After chest injury, air may be introduced into the pulmonary arterial system. If the arterial gas embolism occurs, the smaller arteries or arterioles will be obstructed by air bubbles. This leads to ischemic and hypoxic changes. In addition, air bubbles may induce some inflammatory reactions. Loss of consciousness and convulsion are two of the most severe symptoms that could happen.^2^ CT scan reveals the bubbles in the brain. Hyperbaric oxygen therapy is recognized as a useful way to decrease bubble size and remove the obstruction of vessels.^3^
